# Supplementary material for: Results of a Cluster Randomized Controlled Trial to Promote the Use of Respiratory Protective Equipment among Migrant Workers Exposed to Organic Solvents in Small and Medium-Sized Enterprises
Source: Int J Environ Res Public Health. 2019 Aug 31;16(17):3187. doi: 10.3390/ijerph16173187 (PMC6747133; doi:10.3390/ijerph16173187)
Supplement: Supplementary file 1 [file ijerph-16-03187-s001.pdf]

Table S1 Questions for measuring secondary outcomes

Outcomes	Questions	Answers
Occupational health knowledge	Do you think the following description is correct?	
	1. Organic solvents can cause acute and chronic poisoning.	1=yes, 0=no
	2. Organic solvents can be absorbed into the body through the skin.	1=yes, 0=no
	3. Before contact with organic solvents, you should read the Material Safety Data Sheet to understand the product composition, toxicity, and safety measures to be followed.	1=yes, 0=no
	4. You can wash your hands with organic solvents to remove grease.	1=no, 0=yes
	5. *Kaiyoushui, Baidianyou, Kaijiaoshui, Xibanshui* (Common name for organic solvents products in Chinese) contain substances harmful to health.	1=yes, 0=no
	6. In case of contact with organic solvents, you can rinse the contaminated skin with water.	1=no, 0=yes
	7. Personal protective equipment should be worn at all times in environments where organic solvents are present.	1=yes, 0=no
	8. As long as the protective equipment is not damaged, it can be used during the work.	1=no, 0=yes
	9. Before wearing a protective mask, you should check how it fits your face. Masks that do not completely cover your mouth and nose will not provide protection.	1=yes, 0=no
	10. Personal protective equipment contaminated with organic solvents can be discarded directly.	1=no, 0=yes
Attitude towards RPE utilisation	If RPE is available at the enterprise, I will use it.	
	I will change my RPE when it no longer has any effect.	
	I believe I can properly use RPE when exposed to organic solvents.	1=strongly disagree, 2=disagree, 3= don't agree or disagree, 4=agree, 5=strongly agree
	I believe I can use RPE at all times when exposed to organic solvents.	
	I believe I can meet the enterprise's requirements for using RPE.	
	Use RPF during work can reduce exposure to organic solvents.	
	When exposing to organic solvents, appropriate use RPE could prevent occupational diseases.	
	I would choose not to use RPE because it is uncomfortable.	
	I would choose not to use RPE because it lower the work efficiency.	
Participation in occupational health check-ups	Whether you have taken part in occupational health check-ups during the past six months	1=yes, 0=no

RPE: respiratory protective equipment.

Table S2 Baseline characteristics of the 60 study SMEs in intervention and the control groups

Characteristics	Comprehensive intervention (*n*=20)	Top-down intervention (*n*=20)	Control (*n*=20)	Total (*n*=60)
Industry (*n*)				
Furniture	9	8	6	23
Leather goods	1	2	4	7
Electronic manufacturing	2	2	3	7
Paints and coatings	2	3	3	8
Plastic and plastic cement	2	2	2	6
Others	4	3	2	9
Enterprise size (persons) mean (SD, Min-Max)	88(81, 20-300)	146(124, 20-400)	142(137, 20-400)	125(118, 20-400)
Provide occupational health and safety training (Yes) (*n*)	20	20	20	60
Provide free PPE to employees (Yes) (*n*)	20	20	20	60
Instruct employees in PPE utilisation regularly (Yes) (*n*)	20	19	20	59
Provide regular supervision on PPE utilisation (Yes) (*n*)	20	20	20	60
Organize occupational health check-ups (Yes) (*n*)	18	17	19	54

PPE= personal protective equipment.
